# Regenerative Potential of Granulation Tissue in Periodontitis: A Systematic Review and Meta-analysis

**DOI:** 10.1155/2023/8789852

**Published:** 2023-03-07

**Authors:** Naiwen Tan, Maja Sabalic, Linh Nguyen, Francesco D'Aiuto

**Affiliations:** ^1^UCL Eastman Dental Institute, Periodontology Unit, 21 University Street, WC1E 6DE London, UK; ^2^UCL Eastman Dental Institute, Biomaterials and Tissue Engineering, Royal Free Campus, Rowland Hill Street, NW3 2PF London, UK

## Abstract

**Methods:**

Electronic searches were conducted in five databases including CENTRAL, MEDLINE, EMBASE, Web of Science, and Dentistry & Oral Sciences Source using a combination of MeSH terms and keywords up to 21 June 2022. Human studies including patients aged over 18 years with all forms of periodontitis were included. Following the risk of bias assessment, both qualitative and quantitative analyses were performed.

**Results:**

A total of twelve studies were included in qualitative analysis and six of them in quantitative analyses. The evidence suggested that cells derived from periodontitis granulation tissue have osteogenic, adipogenic, chondrogenic, neurogenic, and angiogenic differentiation abilities as well as immunoregulatory properties. In particular, CD44^+^, CD73^+^, CD90^+^, CD105^+^, and CD146^+^ cells were found widely in granulation tissue whilst the only meta-analysis confirmed that CD90^+^ cells were present in lower numbers within the granulation tissue when compared with healthy periodontal tissue (WMD = −23.43%, 95% CI -30.43 to -16.44, *p* < 0.00001).

**Conclusions:**

This review provided further evidence that granulation tissue from patients with periodontitis can be a potential stem cell source for regenerative therapy.

## 1. Introduction

Periodontitis is a noncommunicable disease affecting the majority of the global population, with 11.2% of individuals suffering from the most severe forms [1]. When untreated, it leads to an inexorable reduction of periodontal tissues, and if left untreated, it ends with the loss of the teeth affected [2]. This is why all steps of the treatment of periodontitis are aimed at resolving gingival inflammation confirmed by clinical improvements in probing pocket depths and clinical attachment levels [3], whilst complete tissue regeneration is not always predictable, and it represents a therapeutic challenge.

Successful regenerative therapy requires an appropriate combination of three key elements: (1) progenitor/stem cells that can create new tissue, (2) a biomaterial or scaffold/matrix to carry these cells, and (3) biological signalling molecules or growth factors that can direct the cells to differentiate and eventually form the desired tissue [4].

Endogenous multipotent stem cells, especially those of mesenchymal origin, have been widely applied in regenerative therapy [5]. These cells have been harvested from various sources including bone marrow aspirate [6, 7], dental pulp [8, 9], or periodontal ligament [10] from extracted teeth, periosteum, gingival connective tissue [11–14], and adipose tissue [15]. Nevertheless, these sources are not always applicable including some cell-isolation protocols carrying greater morbidity than the localized surgical procedure needed.

Granulation tissue is defined as the chronic inflammatory component of periodontal tissues in periodontitis, and it is usually removed during the treatment. This tissue has recently attracted researchers' attention as it contains a large number of multipotent stem cells, and it could be a source of stem cells and matrix for cell-based therapies in regenerative medicine and dentistry [16–18].

This systematic review is aimed at critically appraising all the available evidence on (a) the presence of putative stem and progenitor cell populations in periodontal granulation tissue and (b) the differences between stem cells derived from periodontal granulation tissue when compared to healthy tissue with regard to proportions of cells within the tissue, specific subpopulations, and functional differences.

## 2. Materials and Methods

### 2.1. Protocol and Registration

The protocol of this study was registered on the PROSPERO database, ID number: CRD42021243671, and this review followed the PRISMA 2020 guidelines.

The overall research question was set as follows: “What are the content and functional differences of stem cells in granulation tissue of patients with periodontitis compared to healthy controls?”

### 2.2. Eligibility: Inclusion and Exclusion Criteria

The following PECO outline was used:

*Population*: participants older than 18 years

*Exposure*: diagnosis of periodontitis

*Comparison*: healthy controls

*Outcome*: proportion and characteristics of putative stem cells in gingival tissue

### 2.3. Outcome Measures

The cellular components within the stem cell niche and characteristics in healthy and inflamed gingival tissue were determined as the population and the function of the stem cell from periodontal granulation tissue and healthy gingival tissue. Furthermore, cell profiles were defined by embryonic stem cell markers and mesenchymal stem cells as well as cell functions including osteoblast, adipocyte, and chondrocyte differentiation.

### 2.4. Study Design and Duration

The review included prospective and retrospective studies with participants aged over 18 years diagnosed with all forms of periodontitis. Case-control studies, case series, cross-sectional studies, cohort studies, and nonrandomised and randomised controlled trials were included. Case report studies, reviews, and studies including participants under 18 years, pregnant, or diagnosed with systemic diseases were excluded in this review.

### 2.5. Information Sources and Search Strategy

The search strategy was conducted in five electronic databases including CENTRAL, MEDLINE, EMBASE, Web of Science, and Dentistry & Oral Sciences Source using MeSH terms and free-text terms limited to English language and up to 21 June 2022 with no publication date restrictions. Further, manual searches from reference lists of all included studies and review articles were conducted for missing records.

### 2.6. Selection Criteria and Data Extraction

Two reviewers (NT and MS) screened titles and abstracts independently, and any disagreement was resolved by discussion. Similarly identified manuscripts were screened for inclusion according to the eligibility criteria by two reviewers independently and in duplicates. Disagreements were resolved by discussion and, if necessary, by intervention from a third reviewer (FD).

Data were extracted by two reviewers (NT and MS) independently, including characteristic data, population, exposure (case definition for periodontitis), and outcome (the stem cell components in periodontal granulation tissue and functional analysis of stem cells derived from periodontal granulation tissue). Authors were contacted to provide necessary details when manuscripts lacked information/data (at least twice). Selected studies were pooled for qualitative and quantitative analyses.

### 2.7. Risk of Bias Assessment

Due to the lack of existing guidelines or tools for risk of bias assessment of *in vitro* studies, quality assessment of included articles was conducted independently by two reviewers (NT and MS) using the ARRIVE guidelines 2.0 (Animal Research: Reporting of *In Vivo* Experiments) [19] for *in vivo* studies and the modified ARRIVE guidelines together with CONSORT (consolidated reporting of trials) guidelines for *in vitro* studies, respectively [20] (Supplementary Tables 1-3). A supplementary checklist was also designed for in vitro studies to improve the quality of our review according to the *Cochrane Review* handbook (Supplementary Table 4).

### 2.8. Data Analysis

Descriptive and quantitative methods were used to synthesise the retrieved evidence from included articles. Pooled mean difference and 95% confidence intervals of typical stem cell biomarkers tested by flow cytometry were calculated using random-effects models. The differences in stem cell biomarker expression from granulation tissue and healthy tissue were considered statistically significant at *p* < 0.05. All the statistical analyses were performed using Review Manager (version 5.4).

### 2.9. Publication Bias Assessment

A formal publication bias assessment could not be performed due to the limited number of studies.

## 3. Results

### 3.1. Study Selection

**Figure 1:**
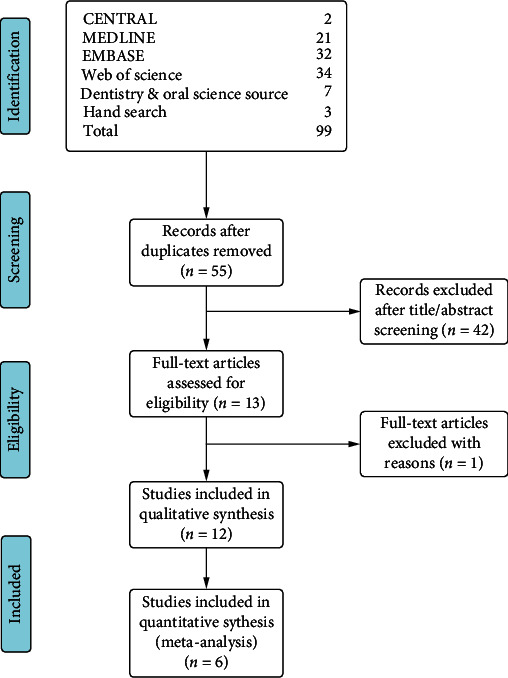
Flow chart of study screening and selection process (following PRISMA guidelines). Nighty-eight studies were retrieved from five databases and hand search, and fifty-four studies were left after the removal of duplicates. Twelve studies were included in qualitative analysis, and six studies were included in quantitative studies after title/abstract and full-text screening.

Ninety-nine eligible articles were retrieved from electronic and hand searches. After removing duplicates, 55 articles were available for title and abstract screening. A total of 13 manuscripts were eligible for full-text assessment, and 12 of them were included in qualitative analysis (one case report article was excluded) (Figure 1). There were 6 case series studies, 5 case-control studies, and 1 randomised controlled clinical trial. Six studies contained flow cytometry analysis data of stem cell biomarkers; hence, they were included in quantitative analysis. The demographic and study design of the studies included in the quantitative analysis was summarized (Table 1).

### 3.2. Bias Assessment

The majority of included studies lacked information on the case definition of periodontitis with only three studies [16, 21, 22] using the old classification (1999), and also, missing information on the demographics of participants and inclusion and exclusion criteria were noted. Out of ten *in vitro* studies, four studies mentioned repeating their experiments whilst only one study reported blinding in evaluation and power calculations (Supplementary Table 5). Combined with the result from the quality assessment checklist for *in vitro* study (Supplementary Table 6), five studies were scored as of fair quality whilst five studies were scored as of low quality. One of the two *in vivo* studies mentioned blinding in data analysis, and the other one gave information on repeating the experiments. Together with the results from quality assessment checklists for *in vivo* studies (Supplementary Tables 7-8), two *in vivo* studies were scored as of fair quality.

### 3.3. Qualitative Analysis

All included studies confirmed the presence of stem or stem-like cells in periodontal granulation tissue. Briefly, 9 articles [17, 22–29] identified mesenchymal stem cells (MSCs), 5 studies [16, 17, 21, 27, 29] identified embryonic stem cell markers, and 3 studies [17, 27, 29] identified hematopoietic stem cells whilst 1 study [25] identified periodontal ligament stem cells (PDLSCs).

### 3.4. Multidifferentiation Ability

Seven included studies [16, 22, 23, 25, 27–29] performed functional analyses including osteogenic, adipogenic, chondrogenic, neurogenic, and angiogenic differentiation of cells isolated from periodontal granulation tissue. Five studies [16, 22, 23, 25, 28] performed cytochemical staining confirming morphological changes and positive reaction to staining. Two studies [25, 28] compared the osteogenic and adipogenic potential between the cells isolated from granulation tissue and healthy oral tissue. One study showed lower osteogenic and adipogenic differentiation abilities of stem cells from granulation tissue when compared with healthy counterparts [28]. Another study found a smaller or reduced total area of mineralised nodules in granulation tissue-derived stem cells compared with healthy tissue-derived stem cells, whilst no difference in adipogenic differentiation potential was observed [25].

Four studies [16, 25, 27, 29] tested relevant gene expressions after induction differentiation. They all showed a marked upregulation of differentiation-relevant gene expression including osteoblastic, adipogenic, neuronal, and angiogenic gene markers. One study tested periodontal ligament- (PDL-) related mRNAs, including scleraxis, periostin, and collagen XII, which were all highly expressed, suggesting that the PDLSCs from granulation tissue could be derived from PDL [25]. One study showed higher expression of ALP gene expression and activity for granulation tissue-derived cells in the osteogenic induction group when compared with the control group [16].

Two studies [22, 25] tested cell differentiation ability *in vivo*. One study [25] transplanted microporous biphasic calcium phosphate carriers subcutaneously into the dorsal of immunodeficient mice loaded with stem cells harvested from inflamed and healthy human PDL. After 8 weeks of healing, newly formed cementum-like and mineralised tissues were observed in the inflamed PDLSC group but were smaller than that in the healthy PDLSC group. Similarly, the other study [22] confirmed the differentiation ability of granulation tissue-derived stem cells in immunocompromised mice, and they found new bone formation when transplanting those stem cells in a critical-sized defect mouse model.

### 3.5. Immunohistochemistry Characterisation

Three studies [22, 25, 26] performed immunohistochemistry on granulation tissue samples. One study showed CD31-stained capillaries, *α*-SMA+ cells (smooth muscle actin), and CD34 staining of the vasculature [26]. Similarly, STRO-1 [22, 25] and CD146 [25] were also stained positively in granulation tissue samples by two different groups.

### 3.6. Proliferation and Migration Ability

Two studies [23, 25] performed proliferation and migration potential assays. One study found a statistically significant higher cell growth rate and a lower migration potential in granulation tissue-derived mesenchymal stem cells when compared with healthy counterparts [23]. The other study concluded a similar proliferative potential between inflamed and healthy PDLSCs but a higher migratory activity in the inflamed PDLSC group [25].

### 3.7. Immunoregulation Ability

Immunoregulatory properties of stem cells isolated from periodontal granulation tissue samples were reported in three studies [17, 28, 30]. Two of them concluded that stem-like cells within inflamed periodontal granulation tissue exhibited similar immunophenotypic characteristics and displayed immunomodulatory properties compared to stem cells from healthy periodontal tissue [17, 30]. A higher expression of TNF-*α*, IL-1*β*, IL-6, and IL-17A was found in granulation tissue than healthy connective tissue confirming the inflammatory state [17] whilst IFN-*γ* showed no statistical significance between the two groups [30]. In contrast, the other study demonstrated that inflamed PDLSCs showed dysfunctional immunomodulatory properties due to diminished inhibition of T-cell proliferation, suppression of Th17 differentiation, and IL-17 production in inflamed periodontal ligament stem cells [28].

### 3.8. Morphological Characteristics

One study [23] performed ultrastructural analysis to observe the cell morphological characteristics of granulation tissue-derived cells by Scanning Electron Microscopy (SEM) and Transmission Electron Microscopy (TEM). MSCs from granulation tissue showed similar ultrastructural characteristics with healthy palate-derived tissue.

### 3.9. MSC Characterization

A Colony-Forming Unit (CFU) assay was performed to demonstrate the presence of MSCs in 3 included studies [22, 23, 25]. Pall and coworkers [23] demonstrated a significantly higher frequency of CFU in healthy palate tissue-derived MSCs than from periodontal granulation tissue-derived MSCs. Park and coworkers [25] found a similar expression of MSC surface antigen (STRO-1^+^, CD146^+^, CD90^+^, CD44^+^, CD19^−^, and CD14^−^) in samples from inflamed and healthy periodontal ligament tissue by the flow cytometry assay.

### 3.10. Embryonic Stem Cell Marker Characterisation

Embryonic stem cell markers were identified in granulation tissue in 6 included studies [16, 17, 21, 27–29], and the multilineage differentiation potential of these tissues was also verified *in vitro*. Ronay et al. [16] found no association between the expression of embryonic stem cell markers and total bacterial loads.

### 3.11. Quantitative Analysis and Meta-analysis

**Figure 2:**
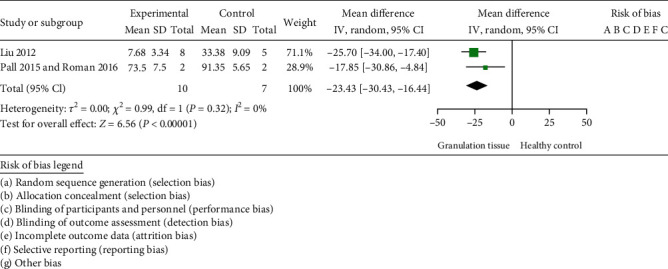
Summary forest plot for the comparison of the expression of CD90 from granulation tissue and healthy control. The random effect, mean difference weighted pooled analysis was used for the assessment. CI: confidence interval.

CD90 expression was evaluated quantitatively in three case-control studies [23, 24, 28] (Figure 2). As two of these studies [23, 24] used tissues from the same single case and healthy control, we analysed their data as a single study. The expression of CD90 from granulation tissue in patients with periodontitis was statistically significantly lower than that from healthy counterparts (MD = −23.43% and 95% CI -30.43% to -16.44%, *p* < 0.00001) (heterogeneity: *p* = 0.32, *I*^2^ = 0%).

**Figure 3:**
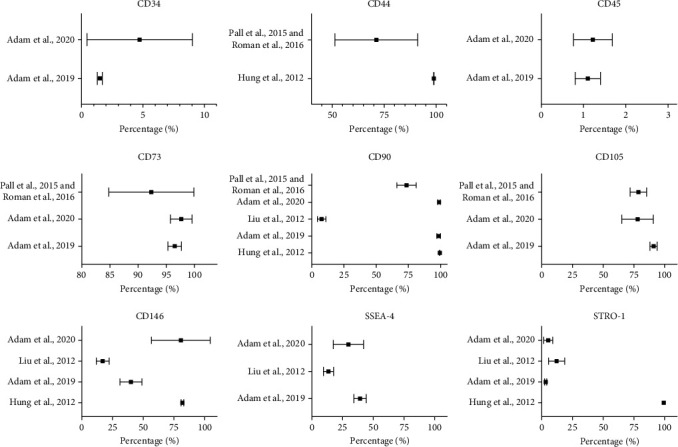
Expression of stem cell biomarkers from included studies by flow cytometry. Mesenchymal stem cell biomarkers (CD44, CD73, CD90, CD105, CD146, and STRO-1), embryonic stem cell biomarker (SSEA-4), and hematopoietic stem cell biomarkers (CD34, CD45) were analysed separately.

The expression of typical stem cell biomarkers in periodontal granulation tissue samples detected by flow cytometry in different articles was then analysed (Figure 3). Two studies [23, 24] were grouped together. A high expression of MSC markers (CD44, CD73, CD90, CD105, and CD146) was reported in most of the included studies but one [28] which described a low level of CD90 and CD146. STRO-1 reduced expression was reported in three studies [27–29] whilst Hung et al. [22] reported the opposite result. A negative expression of CD34 and CD45 was verified by two studies [27, 29], and SSEA-4 showed a medium expression level in three studies [27–29].

## 4. Discussion

This is the first systematic review which confirms that putative stem cells exist in periodontal granulation tissue. Stem-like cells isolated from periodontal granulation tissue exhibited multipotent differentiation and immunoregulatory functions. Whilst the cells isolated from granulation tissue showed typical stem cell biomarkers, they had lower expression of CD90 when compared with healthy counterparts.

MSCs were first isolated from bone marrow and considered precursors of fibroblasts and stromal cells [31, 32]. Bone marrow, however, is not the only reservoir of MSCs, as adult and fetal tissues including muscle [33], adipose [15, 34], dermis [35], periosteum [36], synovial fluid [37], umbilical cord blood [38], and amniotic fluid [39] all contained MSCs. These cells demonstrated trophic [40], anti-inflammatory and immunomodulatory [41–43], antiapoptotic [44, 45], and antimicrobial [46, 47] properties.

Though the identification of multipotent MSCs varies from study to study, there are some common and typical targets detected by most experiments. The International Society of Stem Cell Research proposed three criteria to define MSCs: [48] adherence to plastic; [1] specific surface antigen expression including positive expression (≥95%) of CD105, CD90, and CD73, negative expression (≤2%) of CD34, CD45, CD79*α* or CD19, CD14 or CD11b, and HLA-DR; and [2] multipotent differentiation potential *in vitro* including osteoblasts, adipocytes, and chondroblasts [49]. Considering that CD44, CD73, CD90, and CD105 are normally expressed in fibroblasts and stromal cells, it has further suggested CD146^+^, CD90^+^, CD105^+^, CD73^+^, CD44^+^, Stro-1^+^, CD45^−^, CD11b^−^, and CD14^−^ and little to no expression of CD34 as the plausible phenotype to identify MSCs *in vivo* [32].

All the included studies tested part of typical MSCs or embryonic stem cell biomarkers and most of them appraised the differentiation ability of cells, which can confirm the existence of putative stem cells in periodontal granulation tissue. Thanks to its highly vascularized structure, cells involved, and extracellular matrix components, granulation tissue plays a crucial role during the wound healing process. A high expression of stem cell biomarkers (CD44, CD73, CD90, CD105, and CD146) (Figure 3) suggests a regenerative potential of periodontal granulation tissue harvested from patients with periodontitis and might be an overlooked biological therapeutic factor. Several studies found that the expression of stem cell biomarkers increased with growing cell culture passages [50, 51]. Mitchell et al. [52] found that the expression of typical MSC markers (CD44, CD73, and CD90) increased to over 90% at passage 4 in the adipose-derived mesenchymal stem cell population. One included study [22] showed similar results. The average expression of STRO-1 was 5.91% in the primary tissue section and 6.7% in the primary cell suspension. After culturing for 3 passages, the expression of STRO-1 increased to 99.3%. Further research is needed to understand whether heterogenous fresh cells or granulation tissue could exhibit better therapeutic potential in regenerative medicine.

This review showed a reduced expression of CD90 expression in granulation tissue-derived cells compared with healthy counterparts. This finding is consistent with a study that performed single-cell analysis on human healthy and inflammatory gingival tissues [53]. They identified differences in the composition of cellular subpopulations and found that these changes were relevant to periodontitis progression. Zhao et al. [54] also found that CD90^+^ cells can differentiate into cementoblasts in an experimental model of periodontitis in mice, but this differentiation ability was inhibited by LPS. A similar result was observed by Pelizzo et al. [18] that less efficacious antifibrotic activity was found in granulation tissue-derived MSCs compared with bone marrow-derived MSCs. Future investigations should look out for other stem cell biomarkers between healthy and diseased samples and ultimately get a better understanding of the influence on stem cell components by periodontitis.

The immunoregulatory ability of MSCs is important in their clinical therapeutic application [55]. Long-term inflammation within periodontal granulation tissue unbalances immune response, but whether the immunoregulatory ability of MSCs from granulation tissue is impaired is still unsubstantiated. A previous study showed that a decreased expression of CD90 is also associated with a decreased immunosuppressive activity of MSCs [56]. The immunomodulatory function of granulation tissue-derived MSCs needs further exploration to facilitate their clinical therapeutic application.

**Figure 4:**
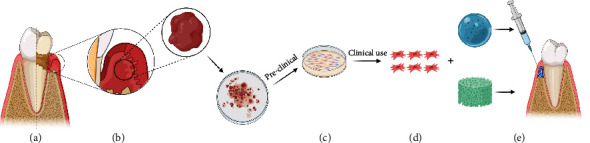
(a) Periodontitis is an inflammatory disease affecting tooth-supporting tissues. Granulation tissue exists in the periodontal defects of patients with periodontitis and is usually discarded in traditional therapy. (b) Granulation tissue can be partially preserved during surgical periodontal therapy or minimally processed, acting as a reservoir of progenitor cells. (c) Heterogeneous cells derived from granulation tissue should be further researched preclinically prior to application in clinical practice. (d) Stem/progenitor cells isolated from granulation tissue might be a potential substitute source of stem cells for cell-based therapies in regenerative medicine and dentistry. (e) A combination of stem cells and different types of biomaterials might be the trend in future clinical practice.

Given their multipotency and multifunction, MSCs may have substantial therapeutic effects *in vivo*, especially in regenerative therapy. Putative MSCs have been found in regenerative periodontal tissues, suggesting that MSCs are crucial in the periodontal regenerative process [57]. Gousopoulou et al. [58] found that putative MSCs also exist in inflamed granulation tissue of peri-implantitis lesions and exhibit multidifferential ability, indicating a regenerative therapeutic potency. Even though these cell-based therapies are still at their exploratory stage, MSCs have already shown promising treatment potential [5, 32]. Because autologous MSCs are limited and can hardly be harvested by a single patient, allogeneic or syngeneic donor cells have been proposed. Despite their immune-privileged property, allogeneic MSCs confer immunological risks as reported in some cases from preclinical animal studies [59] and clinical studies [60]. It is plausible to hypothesize however that harvesting granulation tissue could be an overlooked but promising source of autologous MSCs for regenerative treatments in humans (Figure 4).

In a clinical study, Günay et al. [61] preserved granulation tissue from peri-implant mucosa after nonsurgical therapy, and all 3 patients showed a reduction in probing depth and inflammation. These regenerative tissues kept stable after 2.5 years, 3 years, and 6 years, respectively. The author declared three advantages of preserving intralesional granulation tissue: [48] preserving regeneration essential MSCs, [1] preserving vascular network which benefits wound healing, and [2] preserving the body's matrix which may contribute to preventing a postoperative mucosal recession.

Similarly, Carnevale [62] first reported a novel gingival fibre retention technique in periodontal surgical treatment. According to the surgical procedure, supracrestal attached fibres were preserved whilst the soft tissue not attached to the root surface was removed carefully. The fibre retention technique, which might be including some granulation tissues in preserved fibres, showed a positive outcome in long-term effects in patients with periodontitis in regard to gingival inflammation and tooth loss during supportive periodontal care [63]. More clinical experimental evidence is needed to ascertain whether using these approaches results in clinical benefits in the case of lost periodontal or peri-implant tissues.

## 5. Limitations and Strengths

Some limitations should be emphasized in this systematic review. Though putative stem cell components are demonstrated in periodontal granulation tissue, limited studies and small sample sizes identified urge caution in interpreting the results. Further unclear case definitions and variability in terms of sampling sites limit the validity of the conclusions. Lastly, cells used in experiments were from different passages, which may impact the expression of stem cell biomarkers tested by flow cytometry. This review, however, using a rigorous approach included studies identifying putative stem or progenitor cells by various methods both *in vitro* and *in vivo* supporting our interpretation of the available evidence.

## 6. Conclusions

In conclusion, putative stem or progenitor cells exist in periodontal granulation tissue, and these stem cells show a multidifferential potency *in vitro* and *in vivo*. Further research should focus on exploring how inflammation has an influence on the properties and functions of resident cells in granulation tissue and optimises the regenerative therapeutic ability of granulation-derived stem cells.

## Figures and Tables

**Table 1:** Summary of demographics and study design from studies included in quantitative analysis.

Author, year of publication, and country	Study design	Definition of periodontitis	Ages (*a*: periodontitis, *b*: controls)	*n*, periodontitis/controls	Sample collection site	Biomarkers measured
Pall et al., 2015, Romania	Case-control	Not mentioned	*a* = 39 *b* = 24	1/1	Granulation tissue: removed from the deepest part of surgical area (pocket reduction surgical approach, modified flap operation); healthy control: palatal tissue sample (premolar site), collected during mucogingival surgery (connective tissue graft plus coronally advanced flap)	CD34, CD45, CD49f, CD73, CD90, CD44, CD105, HLA-DR
Roman et al., 2016, Romania	Case-control	Not mentioned	*a* = 38 *b* = 24	1/1	Granulation tissue: removed from the deepest part of surgical area (pocket reduction surgical approach, modified flap operation); healthy control: connective tissue collected during a reentry surgery from the palatal premolar region that was already used as a donor site 6 months before	CD34, CD45, CD49f, CD73, CD90, CD44, CD105, HLA-DR, CD79*α*
Hung et al., 2012, China	Case series	Angular bony defects; chronic periodontitis according to 1999 AAP classification	*a* = 43.33 ± 5.02	15/0	Two- or three-walled intrabony defects	STRO-1, CD146, CD90, CD44
Adam et al., 2019, Germany	Case series	Residual periodontal defect exhibiting a pocket probing depth > 6 mm, bleeding on probing, and a radiographically evident intrabony component of ≥3 mm	*a* = 46.75 ± 2.63	4/0	The bottom of the intrabony periodontal defect	CD73, CD90, CD105, CD146, STRO-1, SSEA-4, CD34, CD45
Liu et al., 2012, China	Case-control	Diagnosed based on marked alveolar bone loss (2/3) and deep probing depth (PD > 5 mm in more than one site)	*a* = 26-40 *b* = 27-45	8/5	Granulation tissue: extracted from tooth surface; healthy control: root apical papilla was gently separated from the surface of the root	CD90, CD146, STRO-1, OCT-4, SSEA-4
Adam et al., 2020, Germany	Case series	Residual periodontal defect exhibiting a probing pocket depth > 6 mm, bleeding on probing, and a radiographically evident intrabony component ≥ 3 mm	*a* = 44.04 ± 5.73	5/0	The bottom of the intrabony periodontal defect	SSEA4, NANOG, SOX2, OCT4, CD90, CD73, CD105, CD146, STRO-1, CD34, CD45

Abbreviation: AAP: American Academy of Periodontology. ^∗^*a* = 26-40, *b* = 27-45, study did not mention exact value but only provided the range of ages.

## Data Availability

The quantitative and qualitative data supporting this meta-analysis are from previously reported studies and datasets, which have been cited. The processed data are available from the corresponding author upon request.

## Additional Points

*Scientific Rationale for the Study*. Our aim was to investigate whether periodontal granulation tissue can be a potential source of stem cells for regenerative therapy. *Principal Findings*. This is the first systematic review confirming that several putative stem or progenitor cells exist in granulation tissue in patients with periodontitis. *Practical Implications*. Periodontal granulation tissue which is usually discarded in traditional therapy could be instead a source of stem cells applied in periodontal regenerative therapy.

## Supplementary Materials

Supplementary Materials The checklists and the results of the quality assessment of included articles are attached in supplementary documents.
