# Supplementary material for: Regenerative Potential of Granulation Tissue in Periodontitis: A Systematic Review and Meta-analysis
Source: Stem Cells Int. 2023 Mar 7;2023:8789852. doi: 10.1155/2023/8789852 (PMC10014158; doi:10.1155/2023/8789852)
Supplement: Supplementary Materials — The checklists and the results of the quality assessment of included articles are attached in supplementary documents. [file 8789852.f1.docx]

**Title:** Regenerative Potential of Granulation Tissue in Periodontitis. A Systematic Review and Meta-analysis

**Authors:** Naiwen Tan, Maja Sabalic, Linh Nguyen, Francesco D’Aiuto

**Appendix**

| **Supplementary Table 1. Checklist used to assess the quality of included *in vitro* studies** | | |
| --- | --- | --- |
| Item | Description | Grade |
| 1 | Title | (0) Inaccurate/nonconcise  (1) Concise/adequate |
| 2 | Abstract: either a structured summary of background, research objectives, key experiment methods, principal findings, and conclusion of the study or self-contained (should contain enough information to enable a good understanding of the rationale for the approach) | (1) Clearly inadequate  (2) Possibly accurate  (3) Clearly accurate |
| 3 | Introduction: background, experimental approach, and explanation of rationale/hypothesis | (1) Insufficient  (2) Possible sufficient/some information (3) Clearly meets/sufficient |
| 4 | Introduction: primary and secondary objectives for the experiments (specific primary/secondary objectives) | (1) Not clearly stated  (2) Clearly stated |
| 5 | Methods: study design explained number of experimental and control groups, steps to reduce bias (demonstrating the consistency of the experiment (done more than once), sufficient detail for replication, blinding in evaluation, etc.) | (1) Clearly insufficient (2) Possibly sufficient (3) Clearly sufficient |
| 6 | Methods: precise details of experimental procedure (i.e., how, when, where, and why) | (1) Clearly insufficient  (2) Possibly sufficient  (3) Clearly sufficient |
| 7 | Methods: How sample size was determined (details of control and experimental group) and sample size calculation. | (1) No (2) Unclear/not complete  (3) Adequate/clear |
| 8 | Methods: Details of statistical methods and analysis (statistical methods used to compare groups) | (1) No (2) Unclear/not complete  (3) Adequate/clear |
| 9 | Results: explanation for any excluded data, results of each analysis with a measure of precision as standard deviation or standard error or confidence interval | (1) No (2) Unclear/not complete  (3) Adequate/clear |
| 10 | Discussion: interpretation/scientific implication, limitations, and generalizability/translation | (0) Clearly inadequate  (1) Possibly accurate  (2) Clearly accurate |
| 11 | Statement of potential conflicts and funding disclosure | (0) No  (1) Yes |
| 12 | Publication in a peer-review journal | (0) No  (1) Yes |

|  |
| --- |

| **Supplementary Table 2**. ARRIVE essential checklist for in vivo studies | | |
| --- | --- | --- |
| **Items** | **Description** | **Grade** |
| 1 | Study design: for each experiment, provide brief details of study design including: a. The groups being compared, including control groups. If no control group has been used, the rationale should be stated. b. The experimental unit (e.g., a single animal, litter, or cage of animals). | (0) Not reported  (1) Inadequately reported  (2) adequately reported |
| 2 | Sample size: a. Specify the exact number of experimental units allocated to each group, and the total number in each experiment. Also indicate the total number of animals used.  b. Explain how the sample size was decided. Provide details of any a priori sample size calculation, if done. | (0) Not reported  (1) Inadequately reported  (2) adequately reported |
| 3 | Inclusion and exclusion criteria: a. Describe any criteria used for including and excluding animals (or experimental units) during the experiment, and data points during the analysis. Specify if these criteria were established a priori. If no criteria were set, state this explicitly. b. For each experimental group, report any animals, experimental units, or data points not included in the analysis and explain why. If there were no exclusions, state so. c. For each analysis, report the exact value of n in each experimental group. | (0) Not reported  (1) Inadequately reported  (2) adequately reported |
| 4 | Randomisation: a. State whether randomisation was used to allocate experimental units to control and treatment groups. If done, provide the method used to generate the randomisation sequence. b. Describe the strategy used to minimise potential confounders such as the order of treatments and measurements, or animal/cage location. If confounders were not controlled, state this explicitly. | (0) Not reported  (1) Inadequately reported  (2) adequately reported |
| 5 | Blinding: Describe who was aware of the group allocation at the different stages of the experiment (during the allocation, the conduct of the experiment, the outcome assessment, and the data analysis). | (0) Not reported  (1) Inadequately reported  (2) adequately reported |
| 6 | Outcome measures: a. Clearly define all outcome measures assessed (e.g., cell death, molecular markers, or behavioural changes). b. For hypothesis-testing studies, specify the primary outcome measure, i.e., the outcome measure that was used to determine the sample size. | (0) Not reported  (1) Inadequately reported  (2) adequately reported |
| 7 | Statistical: a. Provide details of the statistical methods used for each analysis, including software used. b. Describe any methods used to assess whether the data met the assumptions of the statistical approach, and what was done if the assumptions were not met. | (0) Not reported  (1) Inadequately reported  (2) adequately reported |
| 8 | Experimental: a. Provide species-appropriate details of the animals used, including species, strain and substrain, sex, age or developmental stage, and, if relevant, weight. b. Provide further relevant information on the provenance of animals, health/ immune status, genetic modification status, genotype, and any previous procedures. | (0) Not reported  (1) Inadequately reported  (2) adequately reported |
| 9 | Experimental procedures: For each experimental group, including controls, describe the procedures in enough detail to allow others to replicate them, including: a. What was done, how it was done, and what was used. b. When and how often. c. Where (including detail of any acclimatisation periods). d. Why (provide rationale for procedures). | (0) Not reported  (1) Inadequately reported  (2) adequately reported |
| 10 | Results: For each experiment conducted, including independent replications, report: a. Summary/descriptive statistics for each experimental group, with a measure of variability where applicable (e.g., mean and SD, or median and range). b. If applicable, the effect size with a confidence interval. | (0) Not reported  (1) Inadequately reported  (2) adequately reported |

| **Supplementary Table 3**. ARRIVE recommended checklist for *in vivo* studies | | |
| --- | --- | --- |
| **Items** | **Description** | **Grade** |
| 1 | Abstract: Provide an accurate summary of the research objectives, animal species, strain and sex, key methods, principal findings, and study conclusions. | (0) Not reported  (1) Inadequately reported  (2) adequately reported |
| 2 | Background: a. Include sufficient scientific background to understand the rationale and context for the study and explain the experimental approach. b. Explain how the animal species and model used address the scientific objectives and, where appropriate, the relevance to human biology. | (0) Not reported  (1) Inadequately reported  (2) adequately reported |
| 3 | Objectives: Clearly describe the research question, research objectives and, where appropriate, specific hypotheses being tested. | (0) Not reported  (1) Inadequately reported  (2) adequately reported |
| 4 | Ethical statement: Provide the name of the ethical review committee or equivalent that has approved the use of animals in this study, and any relevant licence or protocol numbers (if applicable). If ethical approval was not sought or granted, provide a justification. | (0) Not reported  (1) Inadequately reported  (2) adequately reported |
| 5 | Housing and husbandry: Provide details of housing and husbandry conditions, including any environmental enrichment. | (0) Not reported  (1) Inadequately reported  (2) adequately reported |
| 6 | Animal care and monitoring: a. Describe any interventions or steps taken in the experimental protocols to reduce pain, suffering, and distress. b. Report any expected or unexpected adverse events. c. Describe the humane endpoints established for the study, the signs that were monitored, and the frequency of monitoring. If the study did not have humane endpoints, state this. | (0) Not reported  (1) Inadequately reported  (2) adequately reported |
| 7 | Interpretation/scientific implications: a. Interpret the results, taking into account the study objectives and hypotheses, current theory, and other relevant studies in the literature. b. Comment on the study limitations, including potential sources of bias, limitations of the animal model, and imprecision associated with the results. | (0) Not reported  (1) Inadequately reported  (2) adequately reported |
| 8 | Generalisability/translation: Comment on whether, and how, the findings of this study are likely to generalise to other species or experimental conditions, including any relevance to human biology (where appropriate). | (0) Not reported  (1) Inadequately reported  (2) adequately reported |
| 9 | Protocol registration: Provide a statement indicating whether a protocol (including the research question, key design features, and analysis plan) was prepared before the study, and if and where this protocol was registered. | (0) Not reported  (1) Inadequately reported  (2) adequately reported |
| 10 | Data access: Provide a statement describing if and where study data are available. | (0) Not reported  (1) Inadequately reported  (2) adequately reported |
| 11 | Declaration of interests: a. Declare any potential conflicts of interest, including financial and nonfinancial. If none exist, this should be stated. b. List all funding sources (including grant identifier) and the role of the funder(s) in the design, analysis, and reporting of the study. | (0) Not reported  (1) Inadequately reported  (2) adequately reported |

| **Supplementary Table 4**. Supplementary Risk of Bias Checklist for *In Vitro* studies | | |
| --- | --- | --- |
| **Item** | **Description** | **Grade** |
| 1 | Power calculation/Sample size calculation | Yes/No |
| 2 | Allocation concealment | Yes/No |
| 3 | Repetition/Randomisation | Yes/No |
| 4 | Blinding in analysis | Yes/No |

| **Supplementary Table 5.** Quality Assessment Results of *In Vitro* Studies by Supplementary Risk of Bias Checklist | | | | |
| --- | --- | --- | --- | --- |
| **Studies** | **Power calculation/Sample size calculation** | **Allocation concealment** | **Repetition/ Randomisation** | **Blinding in analysis** |
| Pall et al, 2015 | No | No | Yes | No |
| Roman et al, 2016 | No | No | No | No |
| Ronay et al, 2014 | No | No | No | No |
| Ronay et al, 2013 | No | No | No | No |
| Apatzidou et al, 2018 | Yes | No | No | Yes |
| Li et al, 2014 | No | No | No | No |
| Roman et al, 2015 | No | No | No | No |
| Adam et al, 2019 | No | No | Yes | No |
| Liu et al, 2012 | No | No | Yes | No |
| Adam et al, 2020 | No | No | Yes | No |

| **Supplementary Table 6**. Quality Assessment Results of In Vitro Studies | | | | | | | | | | | | | |
| --- | --- | --- | --- | --- | --- | --- | --- | --- | --- | --- | --- | --- | --- |
| **Studies** | **1** | **2** | **3** | **4** | **5** | **6** | **7** | **8** | **9** | **10** | **11** | **12** | **Overall** |
| Pall et al, 2015 | 1 | 2 | 2 | 1 | 1 | 3 | 1 | 3 | 3 | 2 | 1 | 1 | 21/28 |
| Roman et al, 2016 | 1 | 1 | 2 | 1 | 1 | 3 | 1 | 3 | 3 | 2 | 1 | 1 | 20/28 |
| Ronay et al, 2014 | 1 | 2 | 2 | 2 | 1 | 3 | 1 | 3 | 3 | 2 | 1 | 1 | 22/28 |
| Ronay et al, 2013 | 1 | 2 | 2 | 2 | 1 | 3 | 1 | 1 | 3 | 2 | 1 | 1 | 20/28 |
| Apatzidou et al, 2018 | 1 | 2 | 2 | 2 | 1 | 3 | 1 | 3 | 2 | 2 | 1 | 1 | 21/29 |
| Li et al, 2014 | 1 | 1 | 2 | 1 | 1 | 3 | 1 | 3 | 2 | 2 | 1 | 1 | 19/28 |
| Roman et al, 2015 | 1 | 2 | 2 | 2 | 1 | 3 | 1 | 2 | 3 | 2 | 1 | 1 | 21/28 |
| Adam et al, 2019 | 1 | 2 | 2 | 2 | 1 | 2 | 1 | 3 | 3 | 2 | 1 | 1 | 21/28 |
| Liu et al, 2012 | 1 | 2 | 2 | 2 | 1 | 3 | 1 | 3 | 3 | 2 | 1 | 1 | 22/28 |
| Adam et al, 2020 | 1 | 2 | 2 | 2 | 1 | 3 | 1 | 3 | 3 | 2 | 1 | 1 | 22/28 |

| **Supplementary Table 7**. Quality Assessment of *In Vivo* Studies by ARRIVE Essential Checklist | | | | | | | | | | | |
| --- | --- | --- | --- | --- | --- | --- | --- | --- | --- | --- | --- |
| **Studies** | **1** | **2** | **3** | **4** | **5** | **6** | **7** | **8** | **9** | **10** | **Overall** |
| Hung et al, 2012 | 2 | 1 | 1 | 0 | 1 | 2 | 2 | 1 | 1 | 2 | 13/20 |
| Park et al, 2011 | 2 | 1 | 1 | 0 | 0 | 2 | 2 | 1 | 1 | 1 | 11/20 |

| **Supplementary Table 8**. Quality Assessment of *In Vivo* Studies by ARRIVE Recommended Checklist | | | | | | | | | | | | |
| --- | --- | --- | --- | --- | --- | --- | --- | --- | --- | --- | --- | --- |
| **Studies** | **1** | **2** | **3** | **4** | **5** | **6** | **7** | **8** | **9** | **10** | **11** | **Overall** |
| Hung et al, 2012 | 2 | 1 | 2 | 2 | 0 | 0 | 1 | 2 | 0 | 0 | 2 | Dec-22 |
| Park et al, 2011 | 2 | 1 | 2 | 2 | 0 | 0 | 1 | 1 | 0 | 0 | 2 | Nov-22 |
